# Infection, Replication, and Transmission of Middle East Respiratory Syndrome Coronavirus in Alpacas

**DOI:** 10.3201/eid2206.160192

**Published:** 2016-06

**Authors:** Danielle R. Adney, Helle Bielefeldt-Ohmann, Airn E. Hartwig, Richard A. Bowen

**Affiliations:** Colorado State University, Fort Collins, Colorado, USA (D.R. Adney, A.E. Hartwig, R.A. Bowen);; University of Queensland, Gatton, Queensland, Australia (H. Bielefeldt-Ohmann)

**Keywords:** Middle East respiratory syndrome coronavirus, MERS-CoV, viruses, experimental infection, infection, replication, transmission, immunity, alpacas, respiratory infections, zoonoses, Vicugna pacos

## Abstract

Middle East respiratory syndrome coronavirus is a recently emerged pathogen associated with severe human disease. Zoonotic spillover from camels appears to play a major role in transmission. Because of logistic difficulties in working with dromedaries in containment, a more manageable animal model would be desirable. We report shedding and transmission of this virus in experimentally infected alpacas (n = 3) or those infected by contact (n = 3). Infectious virus was detected in all infected animals and in 2 of 3 in-contact animals. All alpacas seroconverted and were rechallenged 70 days after the original infection. Experimentally infected animals were protected against reinfection, and those infected by contact were partially protected. Necropsy specimens from immunologically naive animals (n = 3) obtained on day 5 postinfection showed virus in the upper respiratory tract. These data demonstrate efficient virus replication and animal-to-animal transmission and indicate that alpacas might be useful surrogates for camels in laboratory studies.

## Abstract 2

These animals might be useful surrogates for camels in laboratory studies of this virus.

Middle East respiratory syndrome coronavirus (MERS-CoV) was first detected in samples from a man in Saudi Arabia who had severe respiratory disease in 2012 (*1*). Since its identification, >1,600 cases of infection have been documented, and the case-fatality rate is ≈36% (*2*). Although efficient human-to-human transmission has been documented, zoonotic spillover probably plays a major role in human infection (*3*–*7*).

Dromedary camels were identified early after recognition of the virus as a possible reservoir host for the disease, although not all patients report contact with camels. Numerous investigators have reported the presence of MERS-CoV RNA or infectious virus in nasal swab specimens of dromedary camels in Saudi Arabia (*3*,*4*,*8*–*10*), Qatar (*5*,*11*–*13*), Oman (*14*), the United Arab Emirates (*15*), Nigeria (*16*), and Egypt (*17*). In some areas of the Middle East and Africa, nearly 100% of animals tested were serologically positive for MERS-CoV, which suggested widespread circulation among camel populations (*9*,*18*,*19*).

Historical samples contained specific antibodies against MERS-CoV as long ago as 1992, which indicated that MERS-CoV has been circulating much longer than originally believed (*19*,*20*). Young animals appear to be at a greater risk for productive infection, and handling practices, such as weaning or shipping animals, might play a major role in animal-to-animal transmission. Many dromedary camels tested had high antibody titers. These results support field data suggesting that young animals become infected, and their immune responses probably are repeatedly boosted by subsequent exposure to the virus (*18*). However, it is currently unknown whether these repeated exposures result in productive infection or whether antibodies generated from a previous infection are protective.

We have previously demonstrated that dromedary camels can be experimentally infected with MERS-CoV and found that mild upper respiratory tract disease associated with shedding copious amounts of virus by nasal secretions develops during the first week after infection (*21*). However, because of the cost of dromedaries, their size, and the requirement for specialized facilities to conduct such studies, it would be useful to identify alternative animal models that respond similarly to infection with MERS-CoV.

We report characterization of an alpaca model of MERS-CoV infection in which we evaluated virus shedding and pathology, transmission by contact, and protective immunity 10 weeks after initial infection. Results indicate that alpacas might be a useful substitute for dromedary camels in certain types of MERS-CoV experiments.

## Materials and Methods

### Ethics

Animal experiments were approved by the Animal Care and Use Committee of Colorado State University. Every effort was made to minimize stress and pain of the animals.

### Virus and Cells

Animals were infected with a low-passage human isolate of MERS-CoV (strain HCoV-EMC/2012). This strain was propagated in Vero E6 cells cultured in Dulbecco modified Eagle medium as described (*21*).

### Animal Study

Nine locally bred alpacas were obtained by private sale for use in this study. Animals were allowed to acclimate to the facility for 1 week before infection and were fed hay ad libitum. One day before infection, animals were subcutaneously injected with an identification and temperature-sensing transponder (Lifechip; Destron Fearing, Dallas/Fort Worth Airport, TX, USA), and their body temperatures were monitored throughout the study. Alpacas A1–A3 were housed together and experimentally infected by intranasal instillation of 10^7^ PFU of MERS-CoV diluted in sterile phosphate-buffered saline (3 mL/nare). Two days later, alpacas A4–A6 were introduced into the same room as alpacas A1–A3 and housed together for the duration of the study.

Nasal swab specimens were collected by inserting and rotating sterile swabs into both nares, immediately placed in virus transport medium, and frozen until assay. Blood was collected weekly into serum-separating tubes for detection of neutralizing antibodies. Animals A1–A6 were held in the facility for 70 days postinfection, and all 6 animals were then reinfected intranasally with 10^7^ PFU of MERS-CoV. Three additional alpacas (A7–A9) were also infected to serve as infection controls and evaluate tissue distribution of virus replication.

Nasal swab specimens were collected daily from all animals for 5 days, at which time animals A7–A9 were humanely euthanized. Tissues collected at necropsy for detection of infectious virus from these 3 animals included nasal turbinates, trachea, larynx, and all 4 lung lobes. These samples plus additional samples, including brain, kidney, liver, skeletal muscle, heart, spleen, bladder, mesenteric lymph node, submandibular lymph node, and mediastinal lymph node, were fixed in formalin for histopathologic and immunohistochemical analysis. Nasal swab specimens and serum samples collected from alpacas A1–A6 were sampled for 2 weeks after the second infection, and then these animals were then humanely euthanized.

### Histopathologic and Immunohistochemical Analysis

Tissues were fixed in 10% neutral-buffered formalin for >7 days and embedded in paraffin. Tissue sections were stained with hematoxylin and eosin and evaluated by a veterinary pathologist (H.B.-O.). Immunohistochemical analysis was preformed to detect MERS-CoV antigen by using a rabbit polyclonal antiserum against HCoV-EMC/2012 antigen (diluted 1:1,000) as a primary antibody as described (*22*).

### Virus Isolation and Plaque Reduction Neutralization Test

MERS-CoV was titrated from nasal swab specimens in virus transport medium and homogenized tissue by plaque assay as described for camels (*21*). A 1-mL volume of virus transport medium was considered a 10^−1^ dilution, and 10-fold serial dilutions were prepared in BA1 medium. Neutralizing antibodies were detected by plaque reduction neutralization test (PRNT) as described, and seropositive animals were identified by using a 90% neutralization cutoff (*23*).

## Results

### Clinical Signs of MERS-CoV in Infected Alpacas

**Figure 1:**
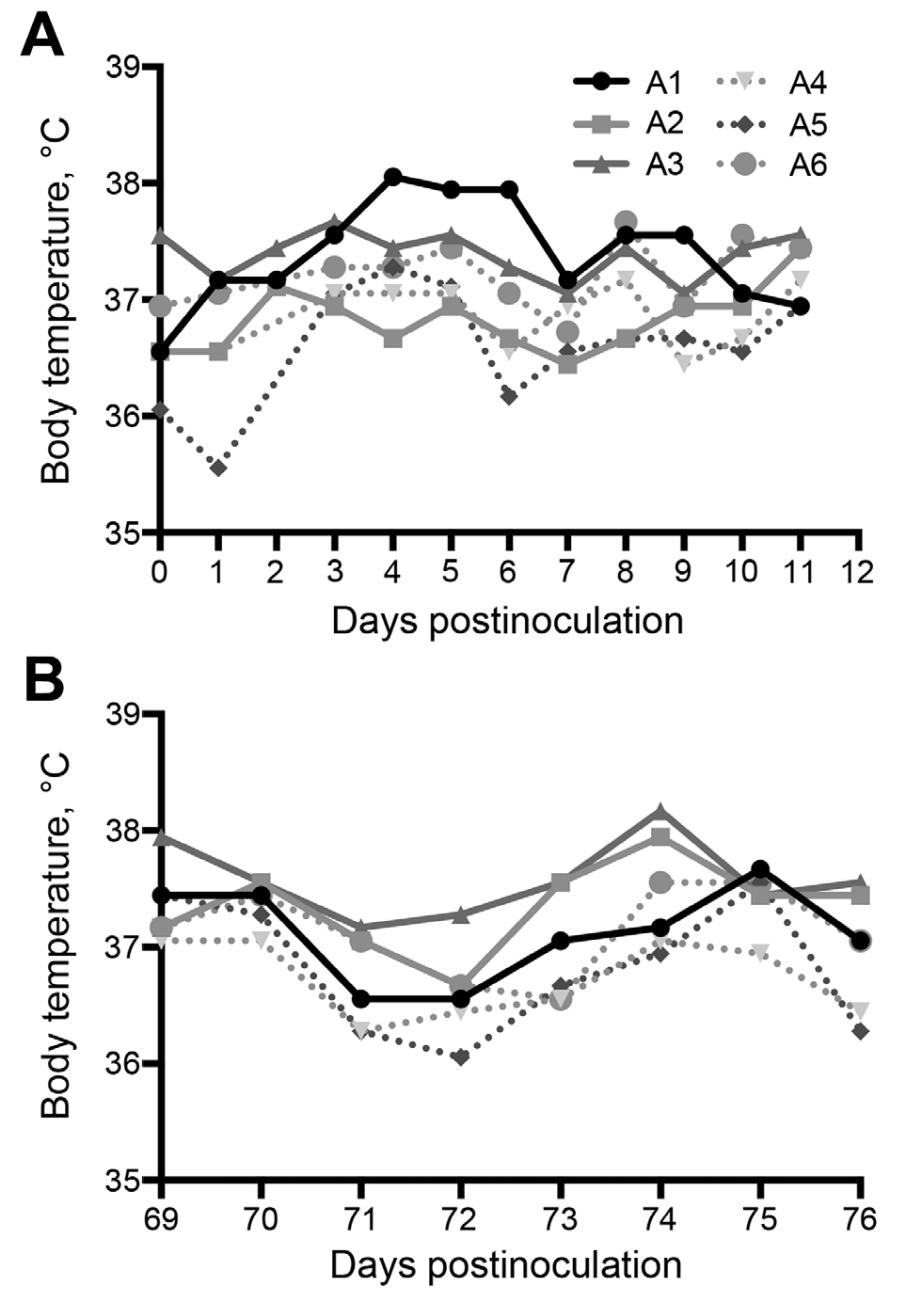
Body temperatures of 6 alpacas (A1–A6) experimentally infected with Middle East respiratory syndrome coronavirus after A) initial challenge and B) after rechallenge on day 70 postinfection.

Field studies and experimental infections suggest that mild respiratory disease associated with nasal discharge develops in MERS-CoV–infected camels (*21*,*24*,*25*). Similar to dromedaries, none of the alpacas had any appreciable increase in body temperature during challenge or rechallenge (Figure 1). Unlike dromedary camels, none of the alpacas had any observable nasal discharge over the course of infection. All alpacas maintained consistent activity level, temperament, and food intake throughout the study.

### Virus Shedding

**Figure 2:**
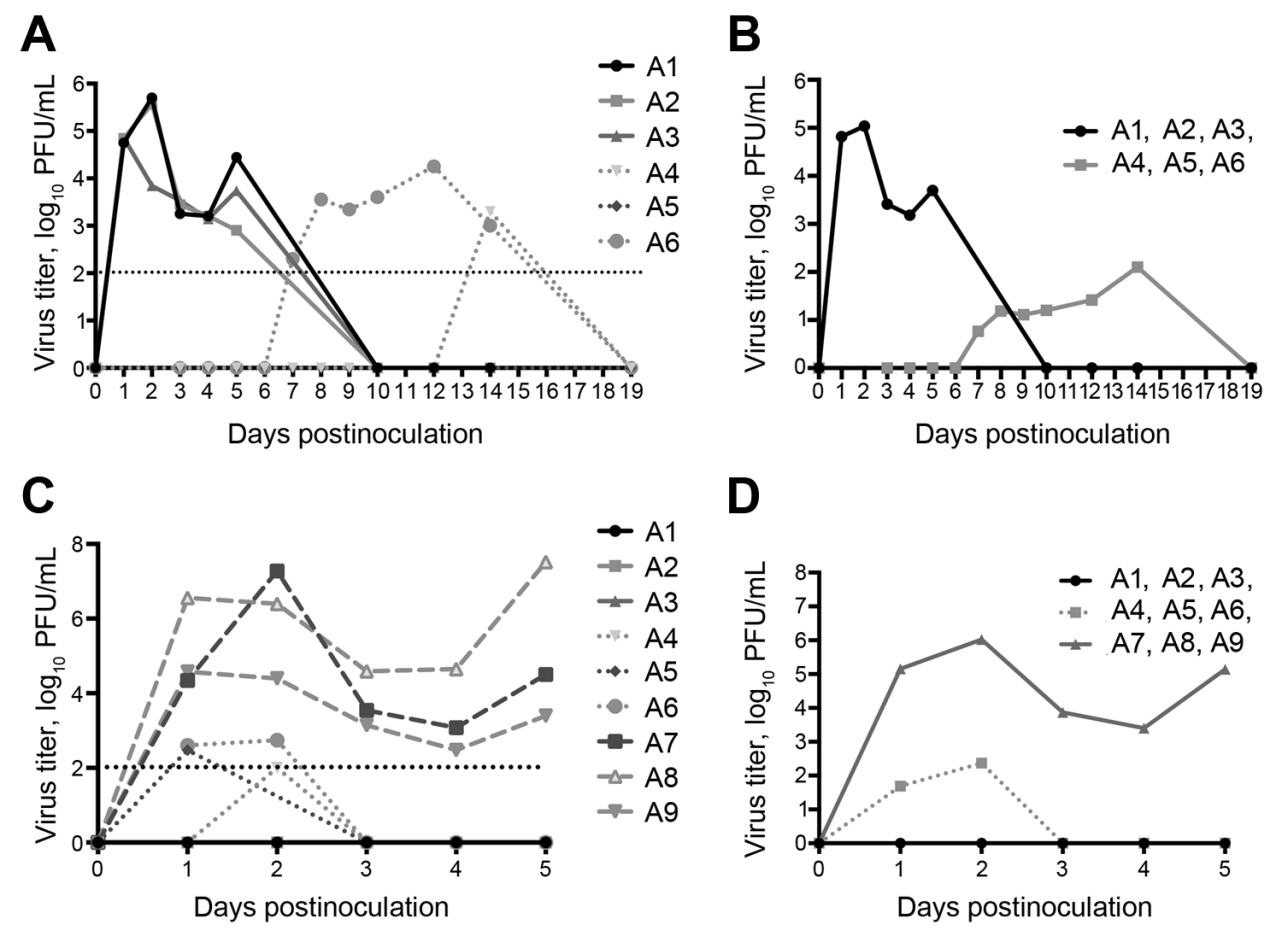
Virus shedding (nasal swab specimens) in 6 alpacas experimentally infected with Middle East respiratory syndrome coronavirus (A1–A3) and co-housed with infected animals (A4–A6). A, B) initial challenge; C, D) rechallenge with addition of 3 immunologically naive alpacas (A7–A9). Individual animal results (A, C) and group means (B, D) are shown. Dotted vertical lines indicate detection limit of the assay.

Nasal swab specimens were collected from infected animals immediately before challenge, on days 1–5 postinfection, and on day 10 postinfection. All 3 experimentally infected animals (A1–A3) had detectable infectious virus on day 1­5 postinfection but had stopped shedding virus by day 10 postinfection (Figure 2, panels A, B). The 3 co-housed animals (A4–A6) were placed in the room with the infected animals 2 days after initial virus infection. Nasal swab specimens were collected from co-housed animals on days 3–10 after infection of animals A1–A3, and then 3 times/week through day 19.

Infectious virus was detected from animal A6 during days 7–14 and from animal A4 only on day 14. We did not isolate infectious virus from animal A5 (Figure 2, panel A). Although infectious virus was detected in animal A6 on day 7, infectious virus was not detected in animal A4 until day 14 (Figure 2, panel A). We speculate that animal A4 became infected by contact with animal A6 after animals A1–A3 had cleared their infections, which suggested that transmission is linked to intimate animal contact, rather than to aerosol transmission.

To test whether previous infection was protective against subsequent virus challenge, all 6 original study animals (A1–A6) were allowed to clear their infections and rechallenged by intranasal infection on day 70 postinfection. Challenge was also performed with 3 immunologically naïve alpacas (A7–A9) (infection controls). The 3 immunologically naive animals became infected and shed virus during days 1–5 postinfection, at which time they were euthanized. The 3 animals that became infected through contact (A4–A6) shed minimal virus between days 1–2 after rechallenge, but not on days 3–5. In contrast, animals that had been experimentally infected were completely protected against rechallenge and did not shed detectable quantities of virus (Figure 2, panels C, D).

### Humoral Response in Infected Alpacas

Serum was collected weekly and tested for neutralizing antibodies against MERS-CoV. All 3 experimentally infected animals (A1–A3) had detectable levels of antibodies beginning on day 14 (Table). Although infectious virus was isolated only from 2 of the 3 co-housed animals, these 3 animals had neutralizing antibodies detected first on day 21 (animals A5 and A6) or day 28 (animal A4) (Table).

**Table:** Neutralizing antibody titers in 6 alpacas after experimental infection with Middle East respiratory syndrome coronavirus and after rechallenge on day 70 postinfection*

Day	Alpaca, antibody titer					
	A1	A2	A3	A4	A5	A6
0	<10	<10	<10	<10	<10	<10
14	40	40	40	<10	<10	<10
21	40	40	40	<10	10	20
28	40	80	80	10	160	20
35	80	160	160	20	80	40
42	160	320	160	20	40	20
49	80	320	80	20	80	80
56	80	640	160	20	80	80
63	80	640	160	40	80	80
70	160	640	80	20	40	80
77	320	640	80	160	320	80
84	320	640	160	320	320	80

*Alpacas A1–A3 were experimentally infected, and alpacas A4–A6 were co-housed with infected alpacas. Titers were determined by using a 90% cutoff.

### Virus in Organs

**Figure 3:**
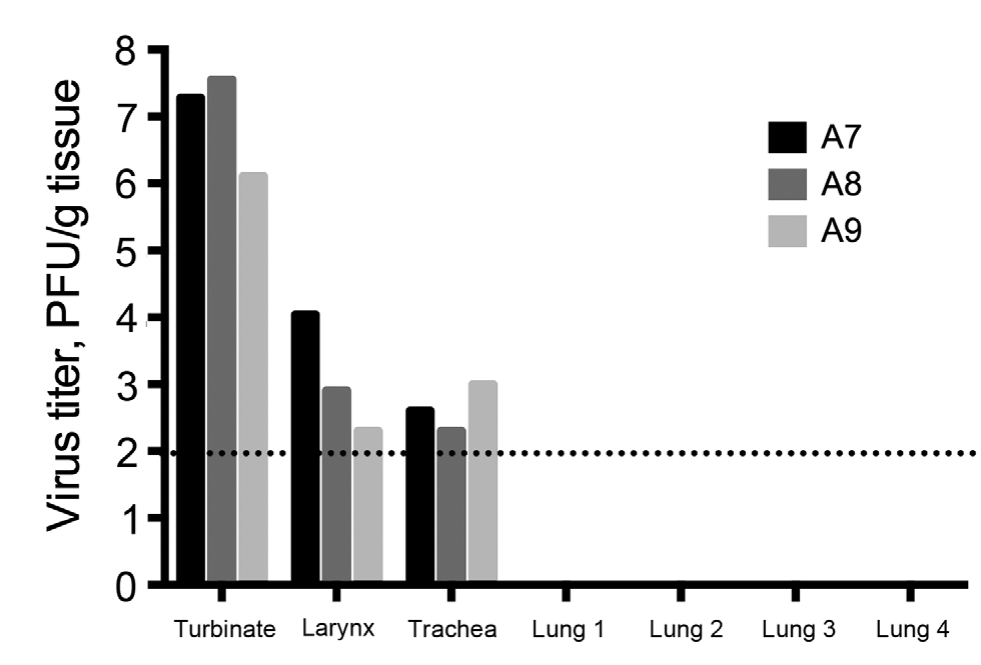
Virus titers from tissues collected from 3 immunologically naive alpacas (A7–A9) challenged with Middle East respiratory syndrome coronavirus and sampled at necropsy on day 5 postinfection. Dotted line indicates detection limit of the assay.

Nasal turbinate, upper trachea, lower trachea, larynx, and all 4 lung lobes were sampled at necropsy from alpacas A7, A8, and A9 and tested for infectious virus by using a plaque assay. Virus was detected in the nasal turbinates, larynx, and trachea of the 3 alpacas but not in any of the lung lobes tested (Figure 3).

### Pathologic and Immunohistochemical Analysis

**Figure 4:**
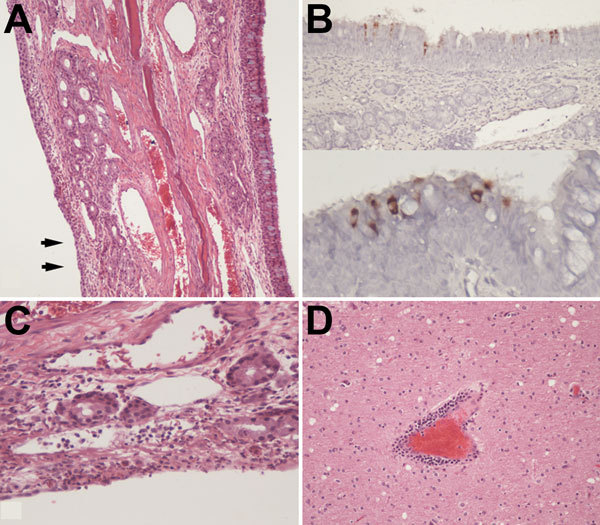
Signs of mild upper respiratory inflammation, encephalitis, and virus antigen detection in respiratory epithelium of alpacas experimentally infected with Middle East respiratory syndrome coronavirus. A) Turbinate from alpaca A8 showing normal respiratory epithelium on the right with goblet cells (blue cells). Epithelium on the left has undergone squamous metaplasia (arrows) and is focally eroded with mild subepithelial inflammation (original magnification ×100). B) Virus antigen in apparently intact respiratory epithelium of alpaca A8 detected by immunohistochemical analysis and lack of subepithelial inflammation (original magnification ×200 [top] and ×400 [bottom]). C) Erosion in turbinate epithelium from alpaca A8 showing leukocytosis in underlying blood vessels (original magnification ×400). D) Perivascular infiltration of lymphocytes and monocytes in the brain of alpaca A9 (original magnification ×200). Hematoxylin and eosin stain in panels A, B, and D; immunohistochemical stain in panel C.

Gross lesions were not observed at necropsy in any of the alpacas. However, microscopic analysis of formaldehyde-fixed tissue sections from animals A7–A9 showed mild squamous metaplasia of the epithelium of the turbinates in animal A8 (Figure 4, panel A) and rare foci of mucosal erosion accompanied by minimal-to-mild subepithelial infiltration of neutrophils and macrophages and fewer lymphocytes (Figure 4, panel C). All 3 animals also had follicular hypertrophy and hyperplasia of the draining lymph nodes, which suggested immune activation.

Immunohistochemical analysis detected rare, scattered, virus antigen–positive cells in respiratory epithelium of turbinates (Figure 4, panel B) and in rare cells interpreted to be intraepithelial leukocytes. Virus antigen was not detected in any of the other tissues examined. Animals A7 and A9 had histopathologic evidence of mild encephalitis with perivascular infiltrates of lymphocytes and monocytes and mild gliosis (Figure 4, panel D). We did not assay brain tissue for virus, either by isolation or PCR, because of the high potential of contamination from the nasal cavity during extraction. Brain tissue was negative for virus by immunohistochemical analysis, but the etiology of the encephalitis observed remains unknown and might have been unrelated to MERS-CoV infection.

## Discussion

Many difficulties are associated with high containment experiments involving dromedary camels. Thus, additional animal models are necessary for MERS-CoV research. Because of their greater availability in the United States and smaller size, we tested an alpaca model.

We report an alpaca model of MERS-CoV infection in camelids and analysis of animal-to-animal transmission and reinfection dynamics. Infected alpacas shed considerable quantities of infectious virus nasally, although at lower concentrations than those reported for dromedary camels (*21*,*24*). In addition, none of the infected alpacas had a noticeable nasal discharge, which is distinctly different from what has been observed in camels and might explain the relatively low efficiency of contact transmission we observed with alpacas.

Infectious virus was detected in nasal swab specimens from 2 of 3 alpacas co-housed with experimentally infected animals, and each of the 3 co-housed animals had neutralizing antibodies against MERS-CoV, which indicated virus transmission. The antibody titers observed approximate those seen for infected dromedaries with the exception of A4, whose antibodies titers remained low until after rechallenge (*21*). Finally, experimentally infected alpacas were completely protected against subsequent virus rechallenge, and contact-infected alpaca were only partially protected.

These results suggest that infection can easily spread among closely grouped camelids infected with MERS-CoV. Camels are frequently moved within the Middle East for grazing, camel shows, and races. Such movement enables mixing and close mingling of animals and could play a major role in MERS-CoV transmission among animals and to handlers. Khalafalla et al. reported that animals bound for slaughter were held in a livestock market for several days, transferred to an abattoir, and kept for up to 24 hours before slaughter (*25*). Our data suggest that these handling practices could promote animal-to-animal virus transmission and that at the time of slaughter virus could potentially be transmitted to slaughterhouse workers.

A major question related to the pathogenesis of MERS-CoV infection in camels, and of great relevance to vaccination strategies, is whether animals that have been infected are resistant to reinfection and virus shedding and, if so, for how long. Our experimentally infected animals were completely protected against rechallenge 70 days later, which suggests that sterilizing immunity can be achieved. However, the animals that were infected through contact (animals A4–A6) shed infectious virus after reinfection, albeit at much lower levels than infected control animals (animals A7–A9).

Although not tested in the present study, it might be surmised that the 3 in-contact animals would have acquired sterilizing immunity from the second (booster) infection. These results support field data that suggest that young animals become infected and probably receive booster infections; most older animals have acquired immunity and are not susceptible to infection and virus shedding (*26*). This finding also highlights the possibility that widespread vaccination of dromedary camels could result in a major decrease in virus transmission to humans.

To date, neutralizing antibodies against MERS-CoV have not been detected in camelids outside Africa or the Middle East. However, if virus were to be introduced into immunologically naive camelid populations, it probably would be readily transmitted among animals. Many New World camelids are valued for their fiber, and such transmission might devastate fiber-related industries (*27*). Thus, as travel-associated cases of MERS-CoV infection continue to be documented, human-to-human virus transmission and possible human-to-animal virus transmission should be monitored.

This study had several limitations. Each of the 3 experimental groups had only 3 animals, which limited our ability to perform statistical analyses. In addition, we evaluated protective immunity 10 weeks after the original infection, which is a relatively short period and does not fully recapitulate seasonal exposures. Thus, further studies are necessary to better understand duration of immunity in camels and alpacas.

**Note Added in Proof:** Crameri et al. also report experimental infection and response to rechallenge of alpacas with Middle East respiratory syndrome coronavirus in this issue of Emerging Infectious Diseases (*28*).
